# Supplementary material for: Chromatin fibers stabilize nucleosomes under torsional stress
Source: Nat Commun. 2020 Jan 8;11:126. doi: 10.1038/s41467-019-13891-y (PMC6949304; doi:10.1038/s41467-019-13891-y)
Supplement: Supplementary file 2 — Supplementary Information [file 41467_2019_13891_MOESM2_ESM.pdf]

Supplementary Information for

”Chromatin fibers stabilize nucleosomes under torsional stress”

Kaczmarczyk et al.

Supplementary Note 1

Nucleosome and tetrasome composition in individual molecules. To ascertain that our samples folded into compact chromatin fibers, we carefully evaluated the composition of individual molecules. Because the force-induced unwrapping of the DNA from nucleosomes results in a significant extension below 10 pN, whereas the second unwrapping transition that is shared by nucleosomes and tetrasomes happens at forces above 10 pN [1], our model can discriminate tetrasomes from nucleosomes. On average, the number of assembled histone complexes slightly deviated from the number of 601-repeats (Fig. 2B and Supplementary Fig. 2B). Despite the selectivity of the 601-sequence, we could not prevent the formation of nucleosomes on the 2 kbp of the flanking non-601 DNA. These additional, randomly positioned nucleosomes may have a lower stability compared to 601-nucleosomes due to the DNA sequence effects and the absence of internucleosomal contacts that would otherwise stabilize the chromatin fiber. As such, their tendency to partially disassemble into tetrasomes is increased. The same likely applies to nucleosomes at the peripheries of the chromatin fibers. Jointly, these effects account for occasional discrepancies between the observed number of nucleosomes and the number of 601-repeats. Either way, by discarding the molecules in which the number of fully folded nucleosomes was less than 90% of the number of 601-repeats, we ensured that the chromatin fiber was the dominant structure in the analyzed tethers.

Supplementary Note 2

Unstacking energy in rotationally unconstrained and constrained fibers. Although we observed that the extension and the stiffness of both 167- and 197-NRL fibers were unaffected by the presence of rotational constraint, nonetheless an increased force was required to achieve the unstacking. This effect derives predominantly from the high torsional stiffness of the DNA handles that need to accommodate the released twist, rather than from inherently stronger interactions between the nucleosomes themselves. As a result, the large linking number stored in the fiber ($\mathrm{Lk_{fiber}}$) cannot freely dissipate (e.g. by swiveling the ends of DNA handles) when a rotationally constrained tether is unfolded. By subtracting the fitted unstacking energy of unconstrained fibers (G_u = 21 $\mathrm{k_BT}$) from that of constrained fibers (G_u = 27 $\mathrm{k_BT}$), we estimated $\sim$ 6 $\mathrm{k_BT}$ of energy required for the over-twisting of DNA handles (Fig. 2B and Supplementary Fig. 2B). In our extended torsional spring model, we uncoupled the contribution of the DNA handles from the entire tether, and obtained a good match between the data and the model with the unstacking energy G_u of 18 $\pm$ 2 $\mathrm{k_BT}$ (mean $\pm$ SD) and 22 $\pm$ 2 $\mathrm{k_BT}$ for 167-NRL and 197-NRL fibers, respectively (Fig. 3C and Fig. 4C), which are consistent with values reported earlier for unconstrained fibers [1]. Overall, our quantitative analysis of chromatin unfolding captures many previously undisclosed aspects of chromatin structure, including the rotational reconfiguration within the fiber.

Supplementary Note 3

Theoretical framework for describing the influence of torque on chromatin tethers. Here we introduce the theoretical framework to describe the influence of torsion on a tether, which consists of a chromatin fiber surrounded by bare DNA handles. Our torsional spring model partitions the linking number change ($\Delta\mathrm{Lk}$) between the fiber ($\Delta\mathrm{Lk}_{\mathrm{fiber}}$) and the DNA handles ($\Delta\mathrm{Lk}_{\mathrm{DNA}}$) that was calculated by imposing equal torque in the entire tether:

$$\mathcal{T} = \mathcal{T}_{\mathrm{DNA}} = \mathcal{T}_{\mathrm{fiber}} \tag{1}$$

By modelling the rotation-extension curves and the torque-rotation curves such that they fit the experimental data, we obtain torsional properties of chromatin fibers.

Model of elastic response of DNA to torsion. The behaviour of bare DNA under torsion can be described by the Worm-Like-Chain (WLC) model with three different, co-existing conformations: twisted, plectonemic and denatured (melted)[2]. We simplify this description by combining the WLC model with one of the dominant conformations of DNA at given linking number density (twisted worm-like-chain, worm-like-chain with plectonemes, or denatured state). Such approximation well captures the properties of DNA at low linking number change, since the coexistence of all three states occurs only in specific force and twist regimes [2] not employed in our experiments.

To describe the extension of DNA under tension and torsion, we derive the free energy of stretched and twisted DNA ($G_{\mathrm{DNA}} = G_{\mathrm{WLC}} + G_{\mathrm{twist}}$) and find the critical linking number change that results in supercoiling or melting of the DNA duplex. The free energy of twisted DNA is

described by a parabolic function:

$$\frac{G_{\mathrm{DNA}}(F,\Delta\mathrm{Lk})}{L}=\frac{G_{\mathrm{WLC}}(F)+G_{\mathrm{twist}}(\Delta\mathrm{Lk})}{L}=-\Big(F-\sqrt{\frac{Fk_{\mathrm{B}}T}{P}}\Big)+\frac{k_{\mathrm{B}}TC_t}{2}\Big(\frac{2\pi\Delta\mathrm{Lk}_{\mathrm{DNA}}}{L}\Big)^2 \quad (2)$$

where L is the contour length of the DNA (in nm), F is the force (in pN), $k_{\mathrm{B}}T$ is the thermal energy (4.1 pN·nm at room temperature), P is the bending persistence length of DNA (set to 50 nm), $\Delta\mathrm{Lk}_{\mathrm{DNA}}$ is the excess linking number of the DNA, C_t is the effective (force-dependent) twist persistence length: $C_t = C_{\mathrm{lim}}(1-\frac{C_{\mathrm{lim}}}{4P}\sqrt{\frac{k_{\mathrm{B}}T}{FP}})$, where C_{lim} is the intrinsic (force-independent) twist persistence length (set to 100 nm).

By taking the derivative of the free energy with respect to force, we can deduce the extension of twisted DNA :

$$\frac{z_{\mathrm{DNA}}(F,\Delta\mathrm{Lk})}{L}=\frac{z_{\mathrm{WLC}}(F)+z_{\mathrm{twist}}(\Delta\mathrm{Lk})}{L}=1-\frac{1}{2}\sqrt{\frac{k_{\mathrm{B}}T}{PF}}-\frac{C^2}{16}\Big(\frac{2\pi\Delta\mathrm{Lk}_{\mathrm{DNA}}}{L}\Big)^2\Big(\frac{k_{\mathrm{B}}T}{PF}\Big)^{\frac{3}{2}} \quad (3)$$

Initially, the excess linking number will induce the DNA to overwind or underwind, leading to the build-up of twist energy. This will generate a torque in the molecule:

$$\mathcal{T}_{\mathrm{DNA}} = C_t\frac{2\pi\Delta\mathrm{Lk}_{\mathrm{DNA}}}{L}k_{\mathrm{B}}T \quad (4)$$

When positive turns are applied and when torque reaches a critical value, known as the buckling torque, the overwound DNA buckles into a plectonemic conformation with a buckling torque:

$$\mathcal{T}_{\mathrm{buck}} = \sqrt{\frac{2k_{\mathrm{B}}TC_p(F-\sqrt{\frac{Fk_{\mathrm{B}}T}{P}})}{1-\frac{C_p}{C_t}}} \quad (5)$$

where C_p is the twist persistence length of plectonemic DNA (set to 24 nm) [3].

By rearranging terms in Supplementary Equation 5, it follows that the maximum excess linking number that can be absorbed by DNA prior to buckling equals:

$$\Delta \mathrm{Lk}_{\max} = \frac{\mathcal{T}_{\mathrm{buck}} L}{2\pi k_{\mathrm{B}} T C_t} \tag{6}$$

Beyond the buckling torque, the DNA coexists in the twisted and plectonemic conformation. The total extension of the DNA decreases linearly with further applied turns according to:

$$\frac{\Delta z_{\mathrm{DNA}}}{\Delta \mathrm{Lk}_{\mathrm{DNA}}} = \frac{2\pi \left(1 - \frac{1}{2}\sqrt{\frac{k_{\mathrm{B}}T}{PF}} - \frac{C^2}{16C_t^2}\left(\frac{k_{\mathrm{B}}T}{PF}\right)^{\frac{3}{2}}\left(\frac{\mathcal{T}_{\mathrm{buck}}}{k_{\mathrm{B}}T}\right)^2\right)}{\frac{\mathcal{T}_{\mathrm{buck}}}{k_{\mathrm{B}}T}\left(\frac{1}{C_p} - \frac{1}{C_t}\right)} \tag{7}$$

As a result, there is an increase of the free energy of plectonemic DNA which equals:

$$\Delta G_{\mathrm{DNA}} = 2\pi \mathcal{T}_{\mathrm{buck}} \Delta \mathrm{Lk}_{\mathrm{DNA}} \tag{8}$$

When negative turns are applied to DNA under low force, it will first underwind and then, at a critical value of torque, it will buckle and form plectonemes, as in the case of positive turns. However, there is an additional critical negative torque, that results in melting (or denaturation) of the DNA duplex. This denaturation torque $\mathcal{T}_{\mathrm{melt}}$, which equals $-10\ \mathrm{pN} \cdot \mathrm{nm}$ [2,4], limits the build-up of twist energy at negative turns. If the calculated buckling torque (Supplementary Equation 5) is smaller than the denaturation torque $\mathcal{T}_{\mathrm{melt}}$, the DNA will coexist in twisted and plectonemic conformations. If the buckling torque is higher than $-10\ \mathrm{pN} \cdot \mathrm{nm}$, the DNA will coexist in two different conformations, twisted and melted. For a small linking number density, we consider

the extensions of melted and twisted DNA to be identical [2]. By rearranging terms in Supplementary Equation 5, it follows that the maximum twist that DNA can absorb prior to melting equals:

$$\Delta \mathrm{Lk}_{\mathrm{max}} = \frac{\mathcal{T}_{\mathrm{melt}} L}{2\pi k_{\mathrm{B}} T C_t} \tag{9}$$

The corresponding free energy gain of melted DNA per turns equals:

$$\Delta G_{\mathrm{DNA}} = 2\pi \mathcal{T}_{\mathrm{melt}} \Delta \mathrm{Lk}_{\mathrm{DNA}} \tag{10}$$

In summary, when turns are applied, the extension of DNA follows the Supplementary Equation 3. Beyond the critical point (defined by Supplementary Equations 6 or 9), this extension reduces linearly with turns (by the factor calculated in Supplementary Equation 7) or remains constant, respectively. Rotation - extension curves modelled with the aforementioned dependencies and the parameters: L = 1900 nm; P = 50 nm; C_{lim} = 100 nm and C_p = 24 nm well describe the experimental data presented in Supplementary Fig. 6.

Model of elastic response of chromatin to torsion. Similarly to DNA, we calculate the energy of the chromatin fiber under torsion. A chromatin fiber can be considered as a Hookean spring with an elastic energy that is dependent quadratically on the change in extension and twist:

$$G_{\mathrm{fiber}} = \frac{1}{2}\frac{c_s}{Nz^0}(\Delta z_{\mathrm{fiber}})^2 + \frac{1}{2}\frac{c_t}{Nz^0}(2\pi \Delta \mathrm{Lk}_{\mathrm{fiber}})^2 + \frac{c_{ts}}{Nz_0}(\Delta z_{\mathrm{fiber}})(2\pi \Delta \mathrm{Lk}_{\mathrm{fiber}}) \tag{11}$$

where N is the number of nucleosomes in the fiber, z_0 is the extension of a single nucleosome (set to 1.5 nm), c_s is the stretch modulus of the fiber per nucleosome (pN·nm^{-1}), c_t is the twist modulus of the fiber per nucleosome (pN· nm^2), c_{ts} is the twist-stretch coupling of the fiber (pN·nm).

The parameter Δz_{fiber} corresponds to the extension change of the chromatin fiber per nucleosome that is induced by force or torque. $\Delta\text{Lk}_{\text{fiber}}$ is the excess linking number of the chromatin fiber per nucleosome.

The linking number change will lead to the build-up of twist energy. This will generate a torque in the molecule:

$$\mathcal{T}_{\text{fiber}} = c_t \frac{2\pi \Delta\text{Lk}_{\text{fiber}}}{N z^0} \tag{12}$$

This dependency allows to infer the torsional stiffness of the chromatin fiber, provided that the linking number change $\Delta\text{Lk}_{\text{fiber}}$ and the torque $\mathcal{T}_{\text{fiber}}$ is known. In the subsequent section, we describe how to quantify these parameters from the experimental data that comprise the dynamics of both chromatin fibers and DNA handles.

Distribution of the linking number between the chromatin fiber and DNA handles. In our experiments, the tether consists of a chromatin fiber that is flanked by ~ 2 kbps DNA handles (jointly referred to below as the 'chromatin tether'). The total excess linking number exerted by twisting the tether must be distributed between the DNA handles and the chromatin fiber (Supplementary Figure 8A). The linking number change in the chromatin fiber is therefore reduced with respect to the total excess linking number (equal to the number of the applied turns) due to the presence of DNA handles (Supplementary Figure 8B):

$$\Delta\text{Lk} = \Delta\text{Lk}_{\text{DNA}} + \Delta\text{Lk}_{\text{fiber}} \tag{13}$$

$$\Delta\text{Lk}_{\text{fiber}} = \Delta\text{Lk} - \Delta\text{Lk}_{\text{DNA}} \tag{14}$$

To calculate how the applied twist is distributed, we combine the Supplementary Equations 1, 4 and 12. It follows that:

$$C_t \frac{\Delta \mathrm{Lk}_{\mathrm{DNA}}}{L} k_{\mathrm{B}} T = c_t \frac{\Delta \mathrm{Lk}_{\mathrm{fiber}}}{N z^0} \tag{15}$$

By rearranging terms in the Supplementary Equation 15, we calculate the twist absorbed by the DNA handles ($\Delta \mathrm{Lk}_{DNA}$). The simplest dependency (excluding twist-stretch coupling) is then:

$$\Delta \mathrm{Lk}_{\mathrm{DNA}} = \frac{L c_t}{N z_f^0 C_t k_{\mathrm{B}} T + L c_t} \Delta \mathrm{Lk} \tag{16}$$

This equation for the excess linking number in DNA handles is then combined with Supplementary Equation 4 to calculate the critical twist that induces supercoiling or denaturation of DNA handles in the chromatin tether. Due to the presence of a soft chromatin fiber, these transitions will occur at a different excess linking number ($\Delta \mathrm{Lk}$) than in the case of a tether with DNA only.

Knowing the distribution of the excess linking number (Supplementary Equations 14 and 16), and the elastic response of DNA handles and chromatin fiber, we can calculate the extension of the chromatin tether (chromatin fiber and DNA handles) as a function of force and the applied turns:

$$z(F, \Delta \mathrm{Lk}, N) = z_{\mathrm{DNA}}(F, \Delta \mathrm{Lk}_{\mathrm{DNA}}) + z_{\mathrm{fiber}}(F, \Delta \mathrm{Lk}_{\mathrm{fiber}}, N) \tag{17}$$

The formula above applies until $\Delta \mathrm{Lk}_{\mathrm{DNA}}$ exceeds the critical value defining the buckling or melting transition. Beyond this point, the extension of the chromatin tether is determined by attributing the applied turns entirely to the dynamics of DNA plectonemes (as in Supplementary Equation 7)

or the denaturing DNA. Note that the plectoneme formation in the presence of histone proteins was calculated with a different persistence length C_p (see Discussion in the main text). In this regime, the excess linking number will lead to the build-up of energy of supercoiled or melted DNA handles, without generating torque build-up in the chromatin fiber. The resulting change in extension follows:

$$z(F, \Delta\mathrm{Lk}, N) = z_{\mathrm{DNA}}(F, \Delta\mathrm{Lk}) \tag{18}$$

This model is applicable at forces below 3 pN when chromatin fiber remains in its compact state. The application of turns at higher tension ($>$ 3.5 pN) results in the chromatin unstacking and hence the extension of the chromatin tether. In the next section, we describe how the statistical mechanics model employed in our earlier work [1] is extended to capture the chirality and the twist-induced unstacking of the chromatin fiber.

Computational determination of twist-induced chromatin unstacking. As shown in our experiments, unstacking of chromatin (induced by either force or twist) yields with an increased tether length and a change in the distribution of the linking number between the fiber and DNA handles. Breaking a single nucleosome-nucleosome contact in a 167-NRL fiber leads to an increase in extension of $\sim$ 76 bp (linker DNA and unwrapped first nucleosomal turn) and $\sim$+0.4 change in linking number (as inferred from the experiments with heparin, see Fig. 1 and Supplementary Figure 1) that must be accommodated by the DNA handles. Additionally, the unstacking comes with its own free energy cost, G_{u}. Here, rather than modelling a two-step unstacking transition as reported previously [1,5], for simplicity, we model the unstacking transition to consist of a single step. We next calculate the free energy of the stretching of the chromatin fiber and take into ac-

count the handedness of the nucleosome and the additional writhe of the linker DNA in the folded chromatin superhelix $\mathrm{Lk_{fiber}}$.

This handedness of the fiber is defined by taking into account the linking number of a single nucleosome and the additional linking number of the linker DNA:

$$\mathrm{Lk_{fiber}} = \Delta\mathrm{Lk_{nuc}} + \Delta\mathrm{Lk_{stack}} \tag{19}$$

where $\Delta\mathrm{Lk_{stack}}$ is the linking number of the linker DNA in the chromatin fiber per nucleosome. The free energy of the fiber is then described by the function:

$$G_{\mathrm{fiber}} = \frac{1}{2}\frac{c_s}{Nz^0}(\Delta z_{\mathrm{fiber}})^2 + \frac{1}{2}\frac{c_t}{Nz^0}\Big(2\pi(\mathrm{Lk_{fiber}}+\Delta\mathrm{Lk_{fiber}})\Big)^2 + \frac{c_c}{Nz^0}(\Delta z_{\mathrm{fiber}})\Big(2\pi(\mathrm{Lk_{fiber}}+\Delta\mathrm{Lk_{fiber}})\Big) \tag{20}$$

We then calculate all probable conformations with the Boltzmann distribution. For a finite number of nucleosomes we can numerically calculate the partition function that includes the energy of the DNA handles and the chromatin fiber:

$$Z = \sum_{\mathrm{i}=0}^{N} \binom{N}{\mathrm{i}} \exp\Big(-\frac{G_{\mathrm{DNA}} + G_{\mathrm{fiber}} + \mathrm{i}G_{\mathrm{u}} - Fz(F, \Delta\mathrm{Lk} + \mathrm{iLk_{fiber}}, \mathrm{i})}{k_{\mathrm{B}}T}\Big) \tag{21}$$

where i is the number of nucleosomes that unfolded, and $\binom{N}{\mathrm{i}}$ is a binomial coefficient that takes the degeneracy of nucleosome unfolding into account [1]. The expected value of total extension at a given force and an excess linking number is then given by:

$$\langle z(F, \Delta\mathrm{Lk}, N)\rangle = Z^{-1}\sum_{\mathrm{i}=0}^{N} z(F, \Delta\mathrm{Lk}+\mathrm{iLk^0_{fiber}}, \mathrm{i})\binom{N}{\mathrm{i}}\exp\Big(-\frac{G_{\mathrm{DNA}} + G_{\mathrm{fiber}} + \mathrm{i}G_{\mathrm{u}} - Fz)}{k_{\mathrm{B}}T}\Big) \tag{22}$$

Supplementary Figures 8B and 8C show how the torsional modulus and the handedness of the chromatin fiber calculated by our model impact the resulting rotation-extension behaviour. The model introduced here was employed to interpret the measured rotation-extension datasets of chromatin fibers and the obtained reproducible results are shown in Supplementary Table 1.

Supplementary Table

nucleosomes	tetrasomes	unstacking energy (k_BT)	stiffness $pN{\cdot}nm^{-1}$	twist modulus $pN\ nm^2$	ΔLk_{stack}
32	8	18	0.8	4	-0.35
32	4	24	1.3	4	-0.35
32	10	21	1.1	4	-0.35
29	2	25	1.2	4	-0.35
30	4	23	0.7	4	-0.3
29	7	25	1.2	3	-0.35
29	7	24	1.2	4	-0.35
29	10	24	1.2	3.5	-0.35
34	13	24	1.2	3.5	-0.35
28	10	20	0.6	4	-0.35

Supplementary Table 1. Parameters from the quantitative analysis of ten individual 167-NRL fibers with a torsional spring model .

Supplementary Figures

Supplementary Figures 1 - 10

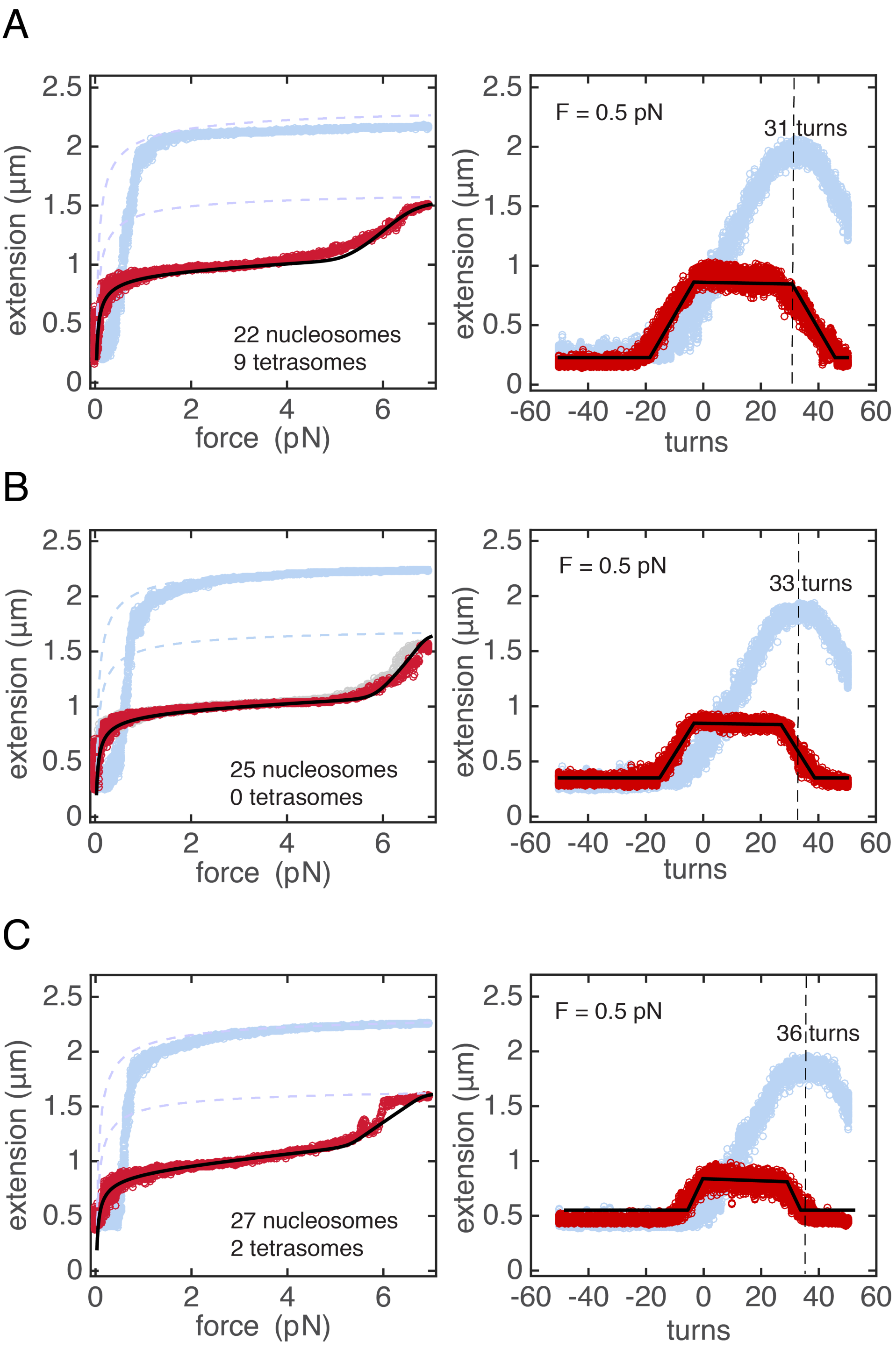

Supplementary Figure 1

Chromatin fiber superhelix stores excessive linking number. Left panels: Force-extension curves of three 167-NRL chromatin fibers (red) with corresponding extensions of deproteinized tethers after treatment with heparin (blue). Black lines are the fit obtained by the statistical mechanics model [1] used to quantify the number of nucleosomes in the fiber ($N_{\text{nucleosomes}}$ = 22, 25, 27 in left panels A, B, C, respectively). Blue dotted lines are the WLC extensions that indicate the length of the unstacked fiber (~1.5 μm) and bare DNA template without nucleosomes (~2.2 μm). The remaining ~0.7 μm of folded DNA would unwrap at higher forces in discrete 25 nm steps (as shown in Fig. 2), revealing at least 28 assembled nucleosomes in studied fibers (700 nm / 25 nm = 28). **Right panels:** Corresponding rotation-extension curves of chromatin fibers (red) shown in left panels, supplemented with rotation-extension curves of the deproteinized tethers (blue). Experiments were performed at 0.5 pN of force. Dotted lines mark the peak of the DNA rotation curve. The shift of the peak with respect to zero twist indicates the linking number that was stored in the chromatin fiber.

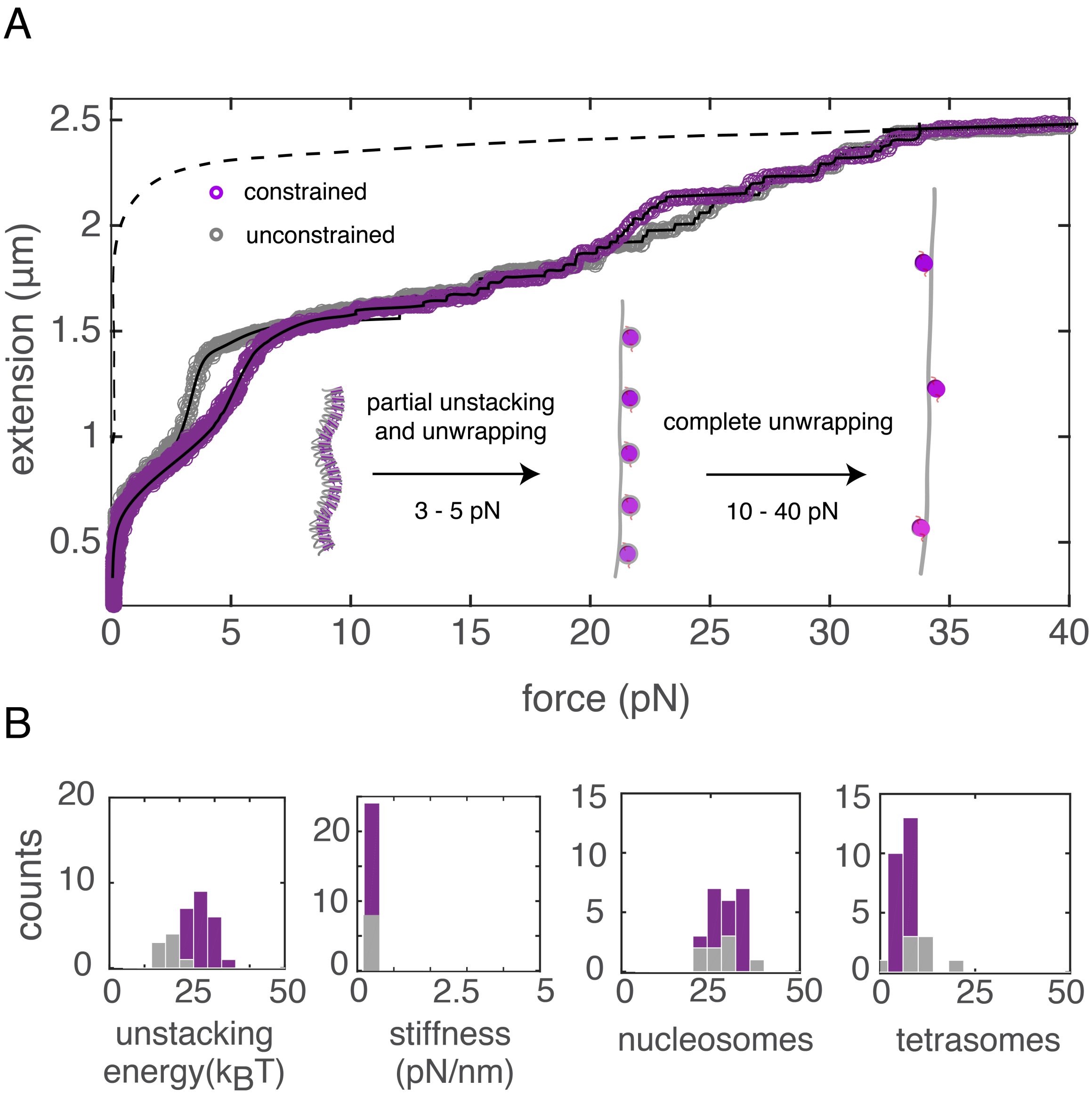

Supplementary Figure 2

Stacking of the 197-NRL fiber is stabilized by torsional constraint. A) A force-extension curve of 197-NRL chromatin fibers assembled on a 601-DNA template that was rotationally constrained (violet) and unconstrained (grey). Both curves were analyzed with the statistical mechanics model [1] (black lines) that yielded with $N_{\text{nucleosomes}} = 31$ and $N_{\text{nucleosomes}} = 32$ for the constrained and the unconstrained fiber, respectively. The extension of bare 7 kb DNA template is plotted with a WLC model (dotted lines). Inset: a cartoon visualizing main conformational changes in chromatin fibers described in the text. **B)** Quantitative analysis of rotationally constrained 197-NRL fibers (n = 24, violet) and unconstrained ones (n = 8, grey) with the statistical mechanics model. The fibers contain on average 30 ± 5 and 29 ± 6 (mean ± SD) assembled core particles, respectively, including nucleosomes and tetrasomes. The stretching stiffness c_s of 0.3 ± 0.1 pN·nm^{-1} and 0.2 ± 0.1 pN·nm^{-1} for constrained and unconstrained fibers, respectively, is similar to values obtained in our previous work [1,5]. The fitted mean unstacking energy G_u of 26 ± 4 k_BT in rotationally constrained fibers is larger than that in unconstrained molecules (17 ± 2 k_BT).

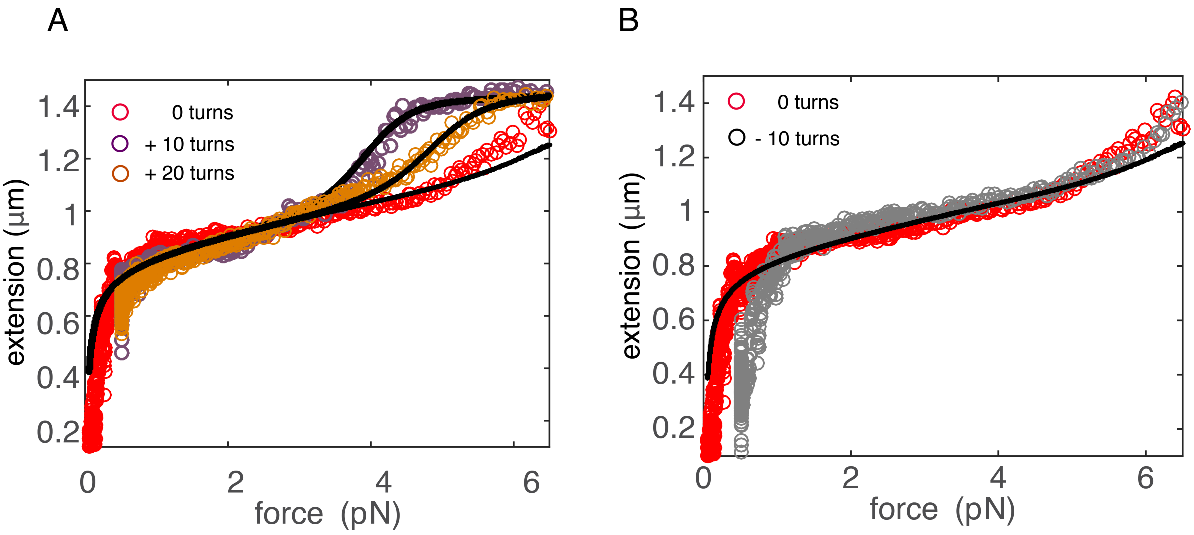

Supplementary Figure 3

Torque regulates the unstacking of the 167-NRL chromatin fiber. Force-extension curves of the 167-NRL chromatin fiber with 20 nucleosomes that were obtained at positive (A) and negative (B) excessive linking number. Solid lines are the fits obtained by the statistical mechanics model. The fiber was pre-twisted, subsequently stretched with low forces to induce its unstacking and then refolded to its initial configuration (refolding curve not shown). The rupture force decreased upon external positive torque reaching the minimum at 3 pN (brown), after pre-twisting with 10 turns. Excessive positive torque changed this tendency and stabilized the fiber. The unstacking transition measured after applying 20 positive turns (orange) required more force than in the preceding stretching cycle. Negative torque (panel B) did not affect the unstacking transition but induced supercoils in the DNA handles.

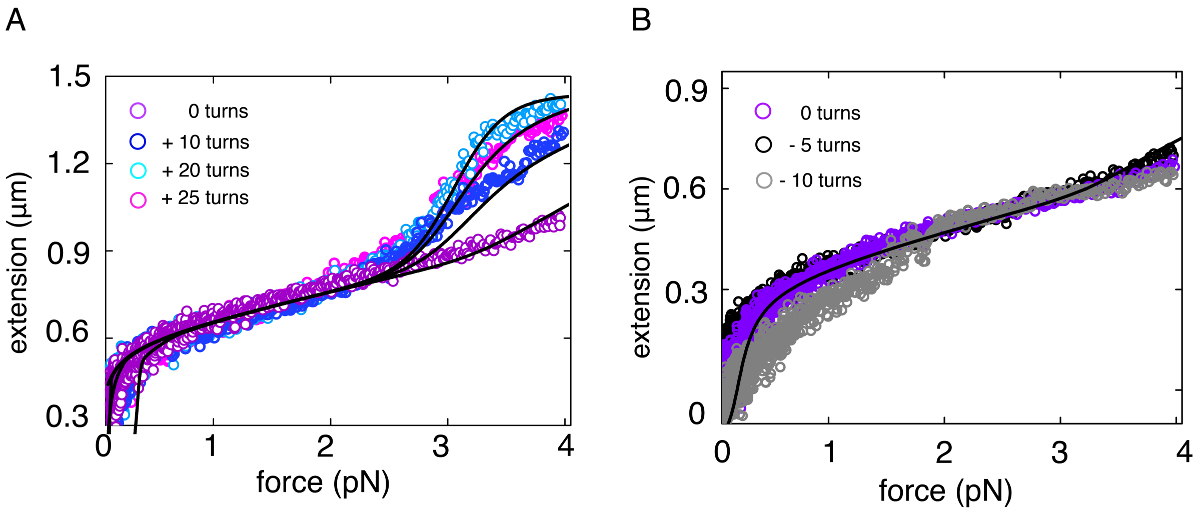

Supplementary Figure 4

Torque regulates the unstacking of the 197-NRL chromatin fiber. Force-extension curves of the 197-NRL chromatin fiber with 25 nucleosomes obtained at positive (A) and negative (B) excessive linking number. Solid lines are the fits obtained by the statistical mechanics model. The fiber was pre-twisted, subsequently stretched with low forces to induce its unstacking and then refolded to its initial configuration (refolding curve not shown). The rupture force decreased upon external positive torque reaching the minimum at 2.5 pN (light blue), after pre-twisting with 20 turns. Excessive positive twist changed this tendency and stabilized the fiber. The unstacking transition measured after applying 25 positive turns (pink) required more force than in the preceding stretching cycle. Negative torque (panel B) did not affect the unstacking transition but induced supercoils the DNA handles.

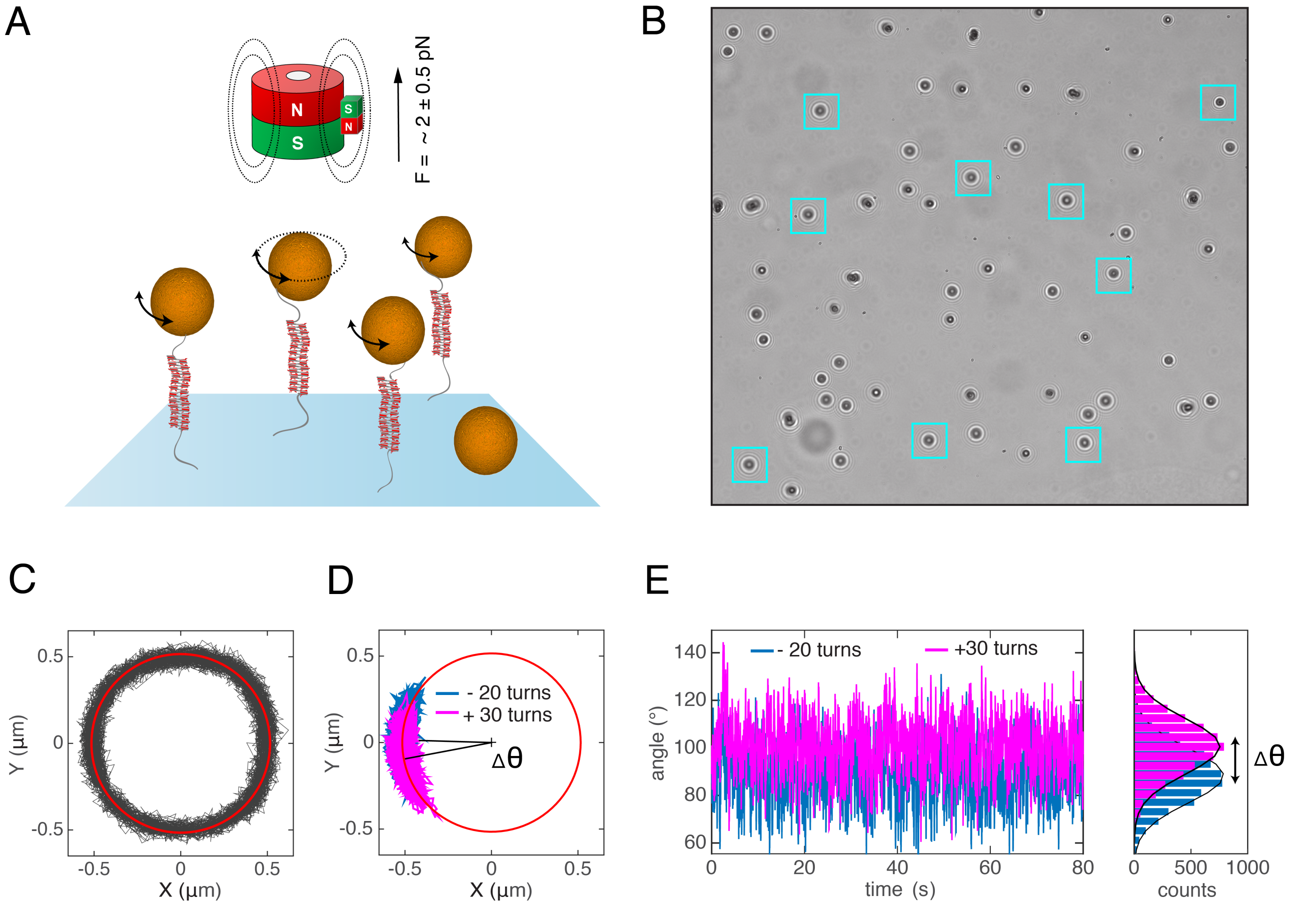

Supplementary Figure 5

Multiplexed magnetic tweezers with a cylindrical magnet configuration enable direct torque measurement on many individual molecules. **A)** Schematic drawing showing molecules stretched with ∼ 2 pN by a cylindrical magnet with a small cubic magnet attached to its side (shown out of scale). Magnetic field lines generated by the cylindrical magnet (dotted lines) are oriented in parallel to the tethers axis. In the absence of the side magnet, a bead that is tethered exactly below the center of the cylindrically-shaped magnet would fluctuate around the tether axis and draw a full circle (dotted circle). Addition of a small side magnet provides with a small transverse field (not shown) that constrains the free rotation of the bead. Additionally, it allows to apply torque to multiple molecules by rotation of the magnet [4], as in classic magnetic tweezers with two cubic magnets. A non-magnetic bead that is attached to the glass surface serves as a reference bead. **B)** A fragment of the field of view with selected tethers for torque measurement. **C)** Tracking of the X, Y position of the tether under rotation. A circle was fitted through the data to obtain its radius. **D)** Fluctuations of the tether in X, Y plane under fixed cylindrical magnet configuration with a small side magnet. Upon application of twist, the tether experience a restoring torque that shifts the arc that represents tether's radial fluctuations. **E)** X, Y fluctuations transformed into polar coordinates to obtain the shift in the angle necessary to quantify the restoring torque.

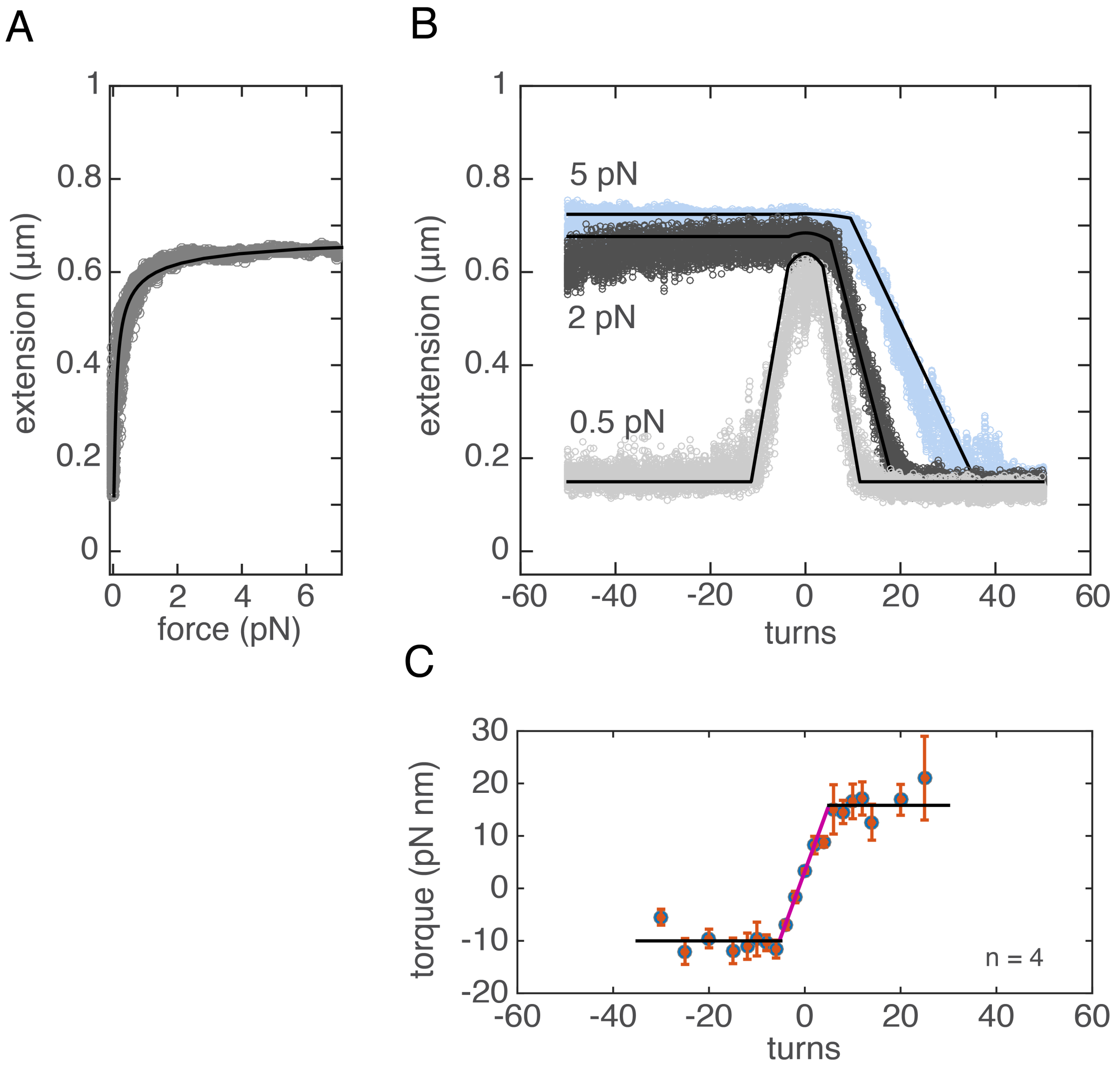

Supplementary Figure 6

Modelling of DNA supercoiling. **A)** A force-extension curve of 1.9 kb DNA molecule fitted to a WLC model yielded a persistence length P = 46 nm. **B)** A rotation-extension curve of the molecule shown in panel A obtained at 0.5 pN (grey) and 2 pN (black) and 5 pN (blue). At low force, the curve is symmetric with respect to zero twist. The extension decreased linearly as the DNA forms plectonemes. At higher tension, negative twist favors DNA melting that does not contribute to a detectable change in the tether's extension. Black lines represent modelled rotation curves with the effective torsional stiffness of DNA C_t = 70 nm at 0.5 pN, C_t = 80 nm at 2 pN, C_t = 83 nm at 5 pN and torsional persistence length of plectonemic DNA C_p = 24 nm. **C)** The averaged torque curve from 4 DNA molecules measured simultaneously at ~ 2 pN. Between -5 and +5 turns, the torque changes linearly. Beyond this regime, the curve remains flat, indicating no torque build-up which occurs upon structural changes such as DNA melting or supercoiling. An independent measure of the torsional stiffness is obtained by fitting the slope of the curve. The fitted C_t = 79 nm (SE = 5 nm) is consistent with the values obtained by quantifying rotation curves. Error bars represent standard deviation of the averaged torque points (n = 4). Source data are provided as a Source Data file.

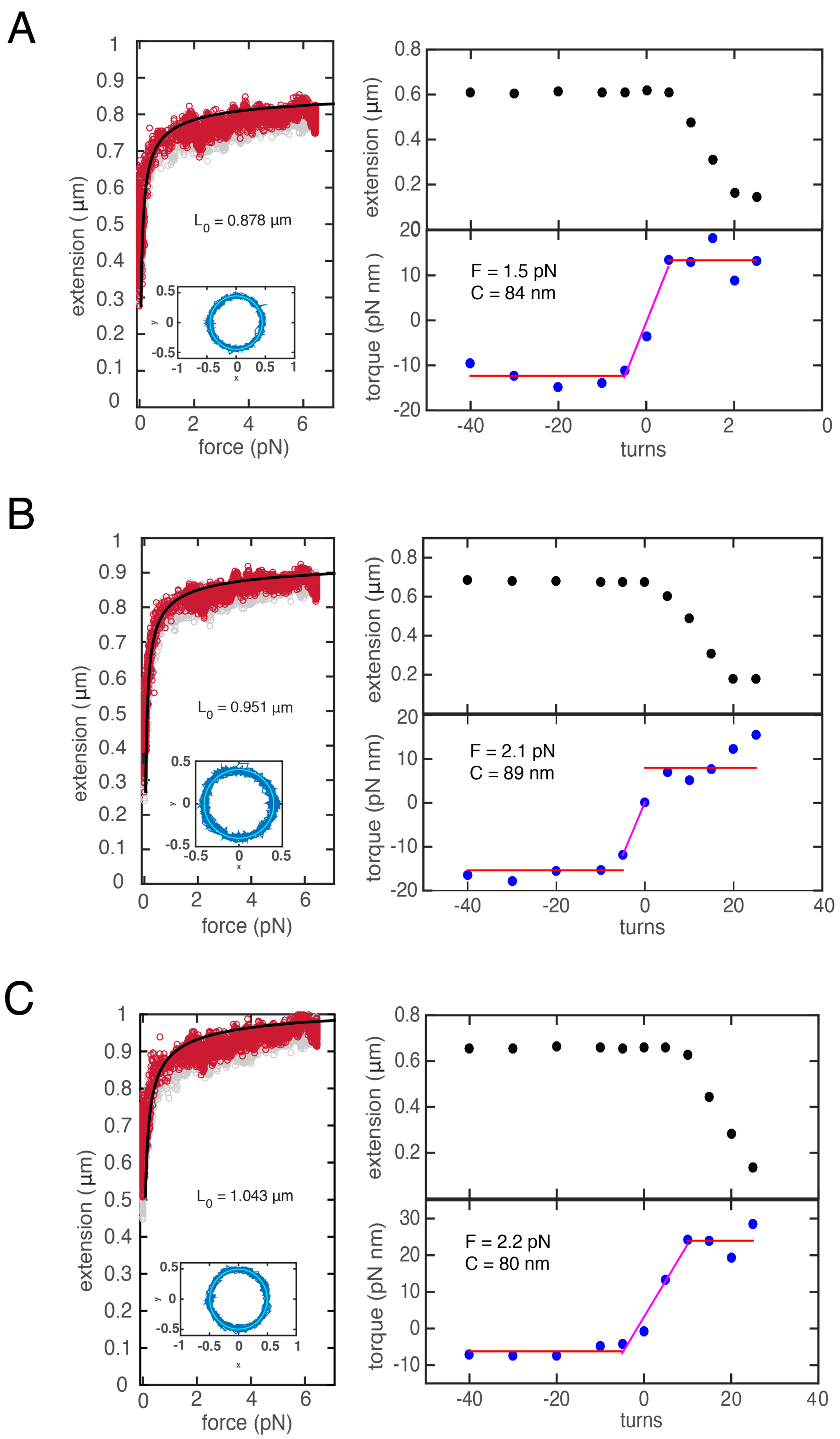

Supplementary Figure 7

Multiplexed torque measurements of DNA molecules. **A-C)** Three representative curves obtained by tracking X, Y fluctuations of multiple tethers under a cylindrical magnet configuration, as in Supplementary Figure 5. Left panels: force-extension curves of the measured DNA molecule obtained with classic MT approach, fitted with a WLC model with the persistence length of 45 nm. Data was offset to account for the "hidden" length of tether due to the off-center attachment to the bead. Inset: X, Y fluctuations obtained upon magnet rotation were used to measure the "hidden length", as in Lipfert *et al.* [4]. Right panels: Torque points (bottom traces) with the corresponding extension measured at this particular measurement point (top traces). The pink line is the linear fit of the slope that is used to quantify torsional stiffness. Red lines indicate the melting and the buckling point.

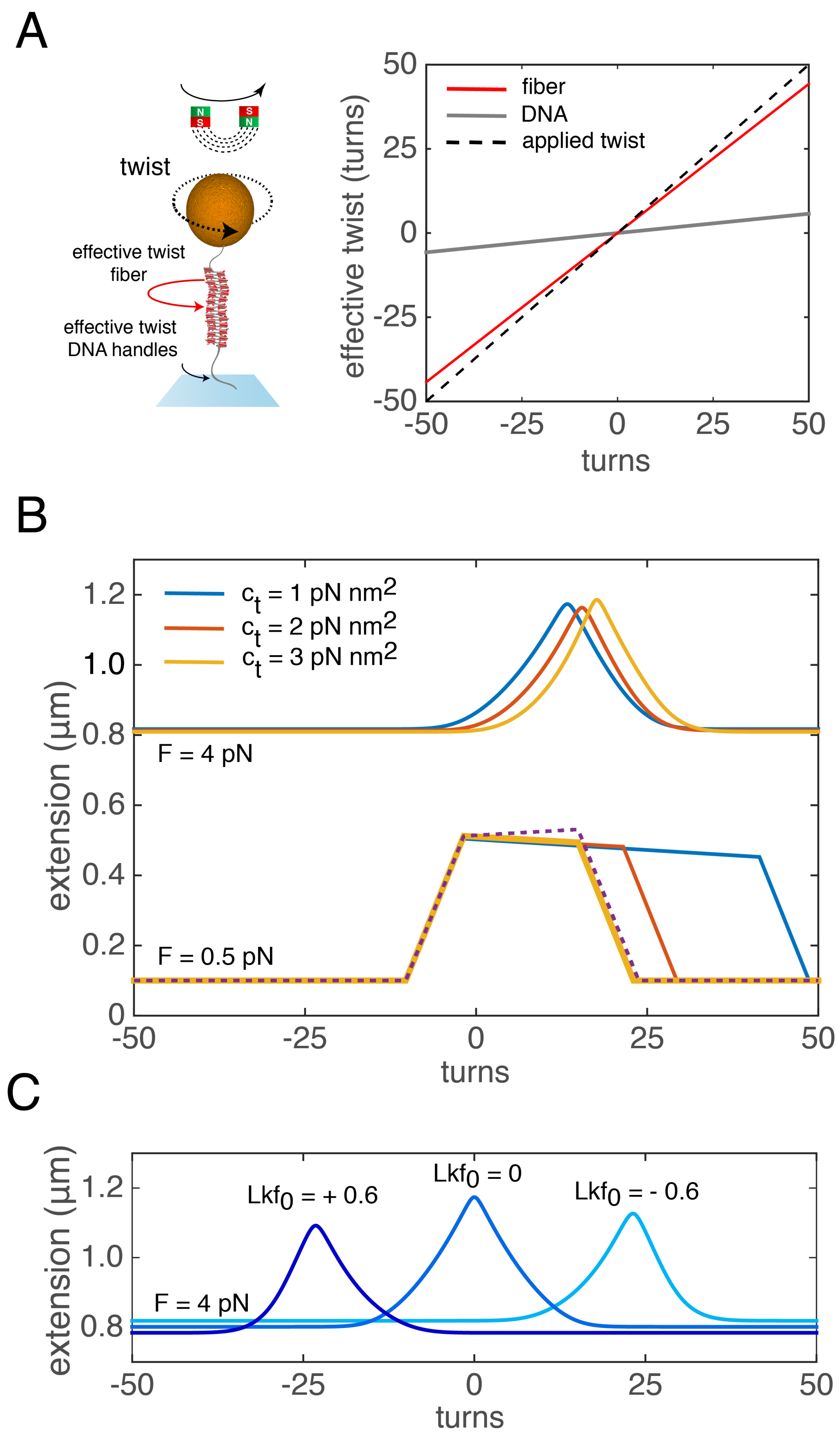

Supplementary Figure 8

The effect of individual parameters in the torsional spring model on rotation-extension curves. **A)** A cartoon of the tethered molecule indicating the distribution of twist between the DNA and chromatin fiber with a plot of the twist build-up in those elements. **B)** A modelled rotation-extension curve of chromatin with 25 nucleosomes with different twist moduli. This parameter affects the buckling point (0.5 pN) and shifts the peak of the curve at 4 pN. The dotted line shows the curve modelled with the same parameters as in the yellow trace, but with a negative twist-stretch coupling factor. **C)** A rotation-extension curve of the same type of fiber as in panel B, modelled with different linking number change upon unstacking.

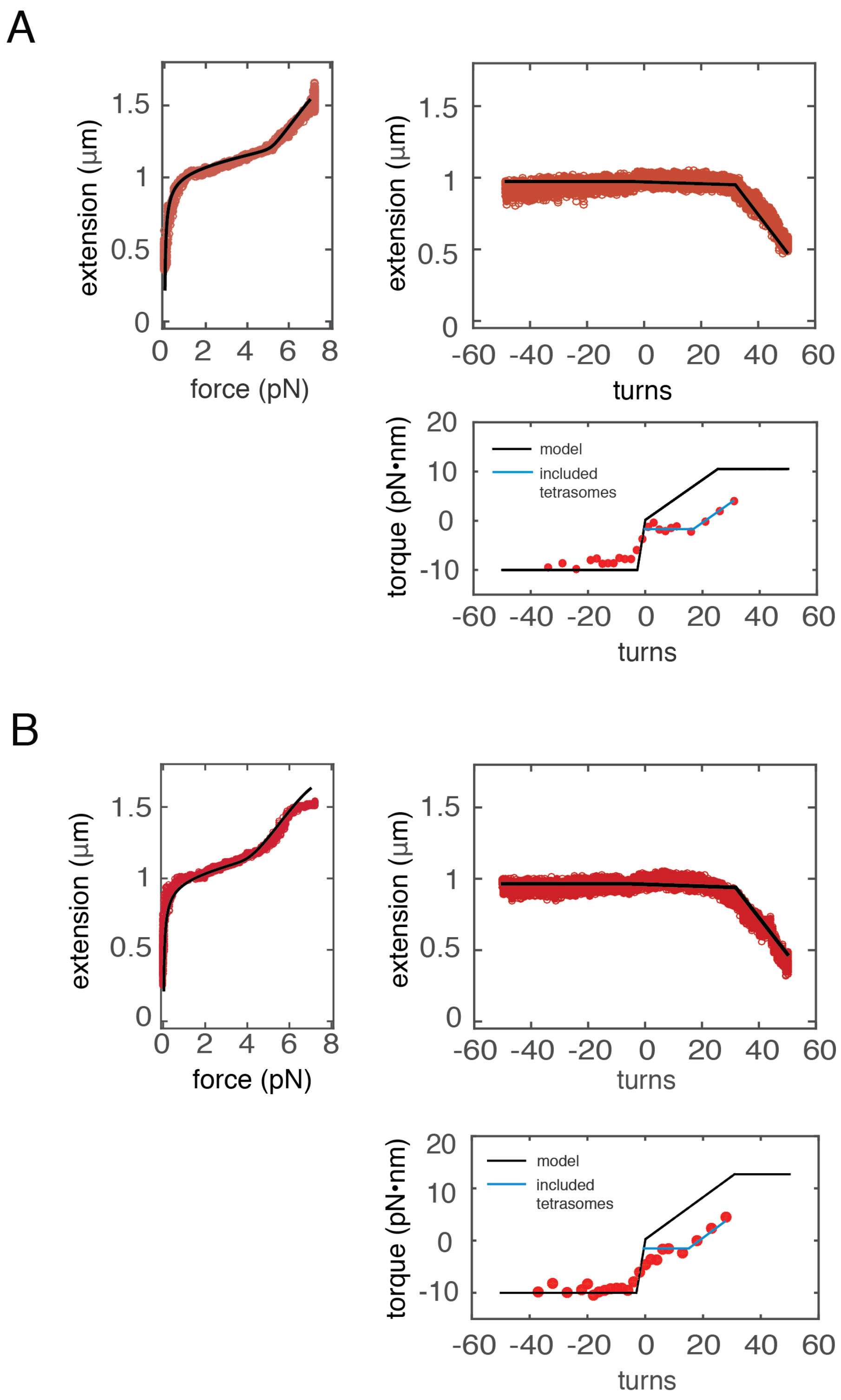

Supplementary Figure 9

Torque measurements in 167-NRL with the corresponding force-extension and rotation-extension curves. A) Selected molecules were analyzed as in Fig. 3. Top left panel shows the force-extension data (red) and the modelled curve (black) with parameters $N_{\mathrm{nucleosomes}} = 24, N_{\mathrm{tetrasomes}} = 2$, unstacking energy $G_{\mathrm{u}} = 22\ k_{\mathrm{B}}T$, stretching stiffness per nucleosome $c_s = 0.85$ pN·nm^{-1}and twist modulus $c_t = 3$ pN·nm^2. Top right panel represents the rotation-extension curve recorded at 2 pN modelled with the same parameters. Bottom panel shows the measured restoring torque as a function of the applied turns. The black line is the prognozed torque from the model. The blue line is the prognozed torque that includes the correction for twist in tetrasomes. **B)** Similar analysis of another molecule yielding parameters: $N_{\mathrm{nucleosomes}} = 24$, $N_{\mathrm{tetrasomes}} = 0$, unstacking energy $G_{\mathrm{u}} = 23\ k_{\mathrm{B}}T$, stretching stiffness per nucleosome $c_s = 0.75$ pN·nm^{-1} and twist modulus $c_t = 3$ pN·nm^2.

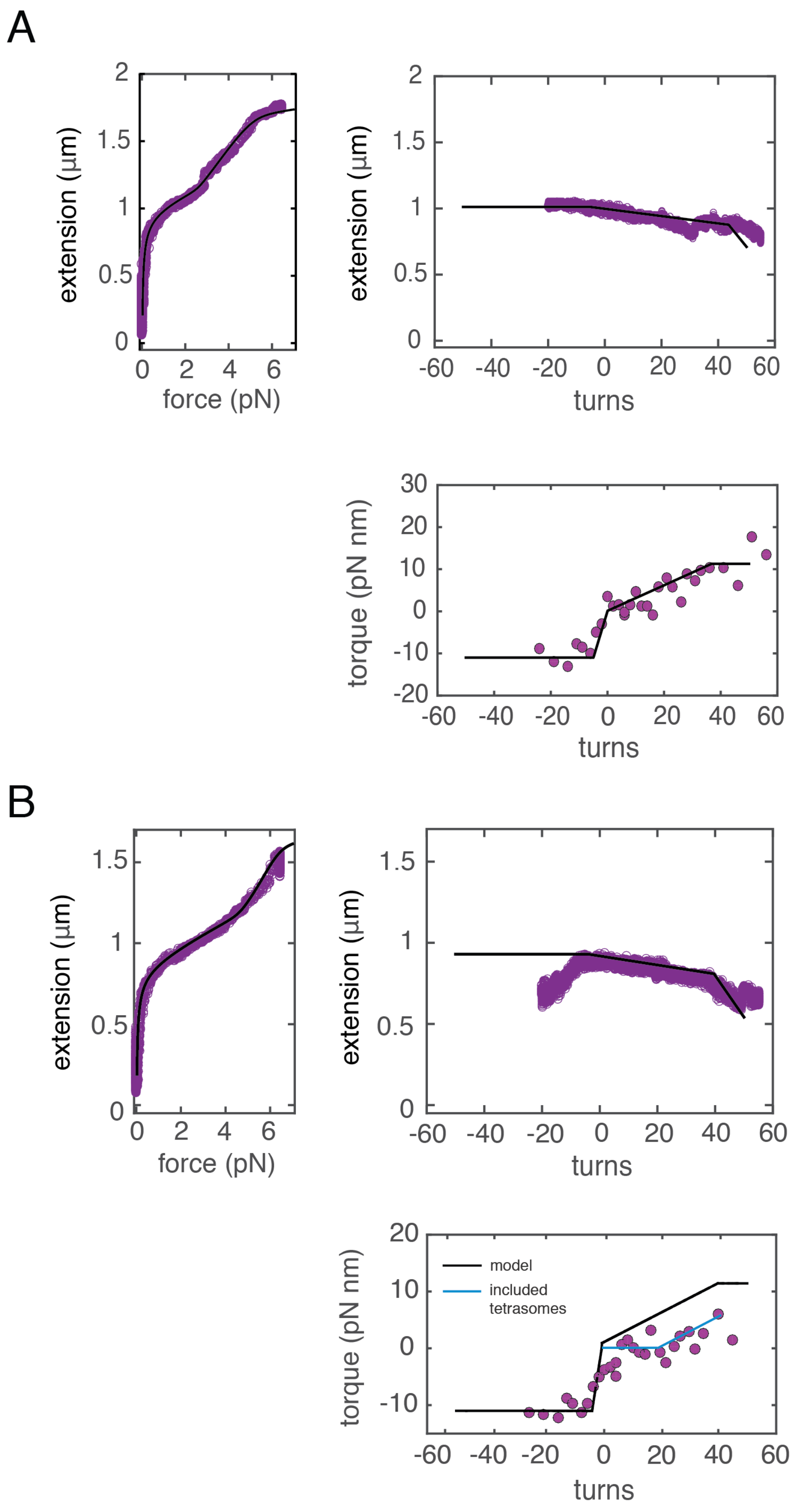

Supplementary Figure 10

Torque measurements in 197-NRL with corresponding force-extension and rotation-extension curves. **A)** Selected molecules were analyzed as in Fig. 4. Top left panel shows the force-extension data (violet) with the modelled curve (black) with parameters $N_{\mathrm{nucleosomes}} = 21$, $N_{\mathrm{tetrasomes}} = 1$, unstacking energy $G_{\mathrm{u}} = 17\ k_{\mathrm{B}}T$, stretching stiffness per nucleosome $c_s = 0.3$ pN·nm^{-1} and twist modulus $c_t = 2$ pN·nm^2. Top right panel represent the rotation-extension curve recorded at 2 pN. Bottom panel shows the measured restoring torque as a function of the applied turns. The black line is the prognozed torque from the model. The blue line is the prognozed torque that includes the correction for twist in tetrasomes. **B)** Similar analysis of another molecule yielding parameters: $N_{\mathrm{nucleosomes}} = 31$, $N_{\mathrm{tetrasomes}} = 15$, unstacking energy $G_{\mathrm{u}} = 18\ k_{\mathrm{B}}T$, stretching stiffness per nucleosome $c_s = 0.4$ pN·nm^{-1} and twist modulus $c_t = 3$ pN·nm^2.

Supplementary References

1. Meng, H., Andresen, K. & Van Noort, J. Quantitative analysis of single-molecule force spectroscopy on folded chromatin fibers. *Nucleic Acids Research* **43**, 3578–3590 (2015).

2. Meng, H., Bosman, J., Van Der Heijden, T. & van Noort, J. Coexistence of Twisted, Plectonemic, and Melted DNA in Small Topological Domains. *Biophysical Journal* **106**, 1174–1181 (2014).

3. Marko, J. F. Torque and dynamics of linking number relaxation in stretched supercoiled DNA. *Physical Review E - Statistical, Nonlinear, and Soft Matter Physics* **76**, 021926 (2007).

4. Lipfert, J., Kerssemakers, J. W. J., Jager, T. & Dekker, N. H. Magnetic torque tweezers: measuring torsional stiffness in DNA and RecA-DNA filaments. *Nature Methods* **7**, 977–980 (2010).

5. Kaczmarczyk, A. *et al.* Single-molecule force spectroscopy on histone H4 tail-cross-linked chromatin reveals fiber folding. *Journal of Biological Chemistry* **292**, 17506–17513 (2017).
