# Supplementary material for: Chromatin fibers stabilize nucleosomes under torsional stress
Source: Nat Commun. 2020 Jan 8;11:126. doi: 10.1038/s41467-019-13891-y (PMC6949304; doi:10.1038/s41467-019-13891-y)
Supplement: Supplementary file 3 — Reporting Summary [file 41467_2019_13891_MOESM3_ESM.pdf]

nature research

Corresponding author(s): John van Noort, Nynke H. Dekker

Last updated by author(s): Jun 8, 2019

Reporting Summary

Nature Research wishes to improve the reproducibility of the work that we publish. This form provides structure for consistency and transparency in reporting. For further information on Nature Research policies, see Authors & Referees and the Editorial Policy Checklist.

Statistics

For all statistical analyses, confirm that the following items are present in the figure legend, table legend, main text, or Methods section.

n/a	Confirmed	
☐	☒	The exact sample size (n) for each experimental group/condition, given as a discrete number and unit of measurement
☐	☒	A statement on whether measurements were taken from distinct samples or whether the same sample was measured repeatedly
☒	☐	The statistical test(s) used AND whether they are one- or two-sided<br>*Only common tests should be described solely by name; describe more complex techniques in the Methods section.*
☒	☐	A description of all covariates tested
☐	☒	A description of any assumptions or corrections, such as tests of normality and adjustment for multiple comparisons
☐	☒	A full description of the statistical parameters including central tendency (e.g. means) or other basic estimates (e.g. regression coefficient) AND variation (e.g. standard deviation) or associated estimates of uncertainty (e.g. confidence intervals)
☐	☒	For null hypothesis testing, the test statistic (e.g. F, t, r) with confidence intervals, effect sizes, degrees of freedom and P value noted<br>*Give P values as exact values whenever suitable.*
☒	☐	For Bayesian analysis, information on the choice of priors and Markov chain Monte Carlo settings
☒	☐	For hierarchical and complex designs, identification of the appropriate level for tests and full reporting of outcomes
☒	☐	Estimates of effect sizes (e.g. Cohen's d, Pearson's r), indicating how they were calculated

Our web collection on statistics for biologists contains articles on many of the points above.

Software and code

Policy information about availability of computer code

Data collection	A C++/CUDA bead tracking algorithm integrated with the LabView 2011 measurement software is available at http://www.github.com/jcnossen/BeadTracker. Datasets presented in Fig. 2, S2, S3, S4 were collected with a custom measurement software written entirely in LabView 2014 and is available upon request.
Data analysis	The analysis of the acquired data was performed using custom-written scripts with built-in functions in Matlab (Mathworks) which are available upon request. Datasets presented in Fig. 2, S2, S3, S4 were analyzed with a custom software written in LabView 2014 which are also available upon request.

For manuscripts utilizing custom algorithms or software that are central to the research but not yet described in published literature, software must be made available to editors/reviewers. We strongly encourage code deposition in a community repository (e.g. GitHub). See the Nature Research guidelines for submitting code & software for further information.

Data

Policy information about availability of data

All manuscripts must include a data availability statement. This statement should provide the following information, where applicable:

- Accession codes, unique identifiers, or web links for publicly available datasets
- A list of figures that have associated raw data
- A description of any restrictions on data availability

The data that support the findings of this study are available from the corresponding author upon request.

Field-specific reporting

Please select the one below that is the best fit for your research. If you are not sure, read the appropriate sections before making your selection.

☒ Life sciences ☐ Behavioural & social sciences ☐ Ecological, evolutionary & environmental sciences

For a reference copy of the document with all sections, see nature.com/documents/nr-reporting-summary-flat.pdf

Life sciences study design

All studies must disclose on these points even when the disclosure is negative.

Sample size	A typical experiment results in ~50 individual chromatin tethers that were measured simultaneously in a multiplexed magnetic tweezer set-up. A subset of these molecules (see Data exclusions) is then analyzed with a statistical mechanics model. The response of a single representative molecule to force/twist is presented in each figure of the manuscript and the result of the quantitative analysis is provided in the figure caption. The results of the quantitative analysis of multiple curves are presented as histograms or tables. The sample size in these is determined by the type of mechanical manipulation exerted on the samples. For stretching experiments of rotationally constrained chromatin tethers (Fig. 2), more than 100 individual molecules from different experiments were analyzed. The sample size is smaller for rotationally unconstrained samples (n=21), as their force-extension relation corresponds with the already published data (doi: 10.1093/nar/gkv215; doi: 10.1074/jbc.M117.791830). A typical twist- and torque experiment results in ~3-5 individual chromatin tethers. In this type of measurement, molecules need to remain intact under longer exposure to a different level of force and torque. Specifically for the torque magnetic tweezers assay, only the molecules tethered close to the center of the camera's field of view can be analyzed, which limits the throughput compared to stretching experiments with canonical magnetic tweezers.
Data exclusions	In all the experiments, multiplexed magnetic tweezers were used to track simultaneously dozens of individual molecules. Among the tethered molecules, a subset had to be excluded from the analysis due to: 1) tethering of more than one DNA-chromatin fiber to a single magnetic bead (detected by a characteristic response to rotation); 2) non-specific attachment (sticking) of some molecules to the surface (detected by sudden large changes in extension upon stretching); 3) lack of rotational constraint (detected as no change in extension throughout the entire rotation cycle); 4) under-saturation or over-saturation of the DNA template with histone octamers (assessed by analyzing the tether extension and fitting to a previously developed statistical mechanics model); 5) too high rotational stiffness of the tether in the case of torque magnetic tweezers measurements (inability to resolve changes in torque).
Replication	Throughout the study, multiple batches of chromatin fibers reconstituted in a bulk assay (salt dialysis) were probed with single-molecule force- and torque- spectroscopy. Each batch of reconstituted chromatin was measured in at least 3 independent series of magnetic tweezers experiments. The resulting force-extension, rotation-extension, and rotation-torque curves were reproducible, taking into account small variations between the composition of the individual chromatin fibers (number of folded histone octamers or tetramers) that could be captured by the quantitative analysis. All experiments were performed in the same buffer conditions, having in mind that different salt concentration would change the folding of chromatin fibers and thereby affect their response to tension and torsion. Similar results were obtained with two magnetic tweezers setups that were operating on a different computing and bead tracking algorithm.
Randomization	Apart from data exclusion process described before, no other experimental groups were further introduced.
Blinding	Blinding was not relevant in this study as there are fixed criteria for allocating the sample to a specific group (a group dedicated for analysis or a group of traces to be excluded).

Reporting for specific materials, systems and methods

We require information from authors about some types of materials, experimental systems and methods used in many studies. Here, indicate whether each material, system or method listed is relevant to your study. If you are not sure if a list item applies to your research, read the appropriate section before selecting a response.

Materials & experimental systems

n/a	Involved in the study	
☐	☒	Antibodies
☒	☐	Eukaryotic cell lines
☒	☐	Palaeontology
☒	☐	Animals and other organisms
☒	☐	Human research participants
☒	☐	Clinical data

Methods

n/a	Involved in the study	
☒	☐	ChIP-seq
☒	☐	Flow cytometry
☒	☐	MRI-based neuroimaging

Antibodies

Antibodies used	Anti-Digoxigenin, from sheep, lyophilizate, Sigma-Aldrich, Cat. No. 11 333 089 001, 200 ug

Validation

The antibody is used for tethering the DNA/chromatin tether to the surface of a flow chamber. If it was not functional, the experiment would not be possible.
